# A screen for protective drugs against delayed hypoxic injury

**DOI:** 10.1371/journal.pone.0176061

**Published:** 2017-04-20

**Authors:** Chun-Ling Sun, Huiliang Zhang, Meng Liu, Wang Wang, C. Michael Crowder

**Affiliations:** 1 Department of Anesthesiology and Pain Medicine, University of Washington School of Medicine, Seattle, Washington, United States of America; 2 Department of Anesthesiology, The Second Military Medical University, Shanghai, People’s Republic of China; 3 Department of Genome Science, University of Washington School of Medicine, Seattle, Washington, United States of America; University of Cincinnati College of Medicine, UNITED STATES

## Abstract

Despite longstanding efforts to develop cytoprotective drugs against ischemia/reperfusion (IR) injuries, there remains no effective therapeutics to treat hypoxic injury. The failure of traditional strategies at solving this problem suggests the need for novel and unbiased approaches that can lead to previously unsuspected targets and lead compounds. Towards this end, we report here a unique small molecule screen in the nematode *C*. *elegans* for compounds that improve recovery when applied after the hypoxic insult, using a *C*. *elegans* strain engineered to have delayed cell non-autonomous death. In a screen of 2000 compounds, six were found to produce significant protection of *C*. *elegans* from delayed death. Four of the compounds were tested in an *ex vivo* mouse heart ischemia/reperfusion model and two, meclocycline and 3-amino-1,2,4-triazole, significantly reduced infarction size. Our work demonstrates the feasibility of this novel *C*. *elegans* screen to discover hypoxia protective drugs that are also protective in a mammalian model of hypoxic injury.

## Introduction

Despite decades of intensive fundamental investigation resulting in numerous clinical trials, no effective treatment has been approved for cytoprotection from ischemia/reperfusion (IR) injury [1–3]. These repeated failures suggest that a full understanding of the mechanisms of IR injury is lacking and that novel, unbiased approaches are needed to make breakthroughs.

A particularly important and vexing question in hypoxic biology is what controls injury after the hypoxic insult and are these factors distinct from those preventing injury before the hypoxic episode. Another fundamental question is the nature of cell non-autonomous injury. How does the injury of one cell promote the death of another nearby or even distant one? This so called “innocent bystander” death is likely to be important in stroke pathology where neurons distant from the ischemic core can undergo delayed death [4]. The difficulty in experimentally manipulating delayed hypoxic cell death and cell non-autonomous death contribute to our incomplete understanding of IR injury.

*C*. *elegans* has been developed as a genetic model to identify and study decisive factors of hypoxic injury [5–10]. Forward and reverse genetic screens in *C*. *elegans* have identified a substantial number of genes (termed Hyp genes), that promote hypoxic injury and whose reduction-of-function phenotype is resistance to organismal death following hypoxia. Utilizing one of the Hyp gene mutants [8], we have recently developed novel transgenic *C*. *elegans* strains where about 3% of non-essential somatic cells were made sensitive to hypoxic injury relative to other cells in the animal. This was accomplished by expressing a wild type version of a Hyp gene in a few cells in the background of the Hyp mutant where all other cells are resistant [11]. We demonstrated that these strains had both delayed cell death and cell non-autonomous death after a hypoxic insult. Thus these strains provide a genetically tractable model to study delayed and cell non-autonomous hypoxic injury and offer tools to screen for genetic changes and chemicals that are protective against delayed hypoxic injury.

Here, we report a screen for drugs to protect from delayed hypoxic organismal death and obtained six compounds that provide reproducibly significant protection in our *C*. *elegans* model. Four of the drugs were tested in a mouse perfused-heart model and two were found to be protective, suggesting that the *C*. *elegans* model may be generally useful to identify compounds protective in various IR injury paradigms.

## Materials and methods

### *C*. *elegans* strains and culture methods

*C*. *elegans* strains were cultured and maintained at 20°C, unless otherwise indicated, on NGM agar with OP50 *E*. *coli* food unless otherwise noted [12]. The N2 (Bristol) strain was the standard wild-type strain from the *C*. *elegans* Genetics Center (CGC, University of Minnesota). Focal hypoxic injury model strains, *rars-1(gc47);gcIs3* and *rars-1(gc47);gcSi4* were previously characterized [11]. The mitochondrial UPR reporter strain SJ4100 (*zcIs13*[*hsp-6p*::GFP]) was obtained from the CGC.

### Hypoxic incubations

Synchronized young adult worms were subjected to hypoxia as described previously except that hypoxic incubation temperature was 26.5°C [9,11]. Briefly, each plate of worms was washed into one 1.5 ml tube with 1 ml of M9 buffer (22 mM KH_2_PO_4_, 22 mM Na_2_HPO_4_, 85 mM NaCl, 1 mM MgSO_4_). Worms were allowed to settle by gravity, and 900 μl of M9 was removed. The tubes were then placed in the anaerobic chamber (Forma Scientific) at 26.5°C for incubation times ranging from 14–27 hours as indicated. Oxygen tension was always ≤ 0.3%. Following the hypoxic insult unless otherwise noted, worms were placed using glass Pasteur pipettes onto NGM plates spotted with OP50 bacteria and recovered at 20°C for the indicated time. Normoxic incubations were otherwise identical except performed in a 26.5°C room air incubator. To score delayed death in the wild type strain N2, hypoxia was performed for 14–18 hours followed by a 24 hour recovery at 20°C; For *gc47;gcIs3* and *gc47;gcSi4*, 27 hours of hypoxia was used followed by 4 days recovery at 20°C and phenotyping.

### Chemical library screen

The Spectrum Collection chemical library from MS-Discovery Inc (Gaylordsville, CT) was supplied through the University of Washington Quellos High Throughput Screening Core. Synchronized young adult *gc47; gcIs3* animals were washed off the NGM/OP50 plates with M9 and washed two times with S-medium [12], and re-suspended in S-medium at approximate 800–1000 worms/ml. 3 ml of the worm suspension was transferred into a 3.5 cm petri dish with addition of 20 μl OP50 (100 mg/ml). The petri dish was covered with a breathable sealing film (Alpha) to prevent evaporation and subjected to hypoxia incubation (O_2_<0.3%) at 26.5°C for 27 hours. Immediately after hypoxic incubation, worms were robotically dispensed into 384-well plates, and the test compounds were added into each well by the robot. The drug solvent of S-medium with DMSO (0.1%) and doxycycline (10 μM) [13] were added to a few wells on each plate as a solvent/negative and a positive controls, respectively. The worms were then allowed to recover in 384-well plates with the various drug solutions in a normoxic incubator at 20°C for 96 hours. Preliminary screens found the death rate under these conditions to be about 70% with no significant death after a normoxic incubation. Before manual scoring of the hypoxic survival, the 384 plates were vortexed for 2 minutes to stimulate the worms. Survival rates of test compounds better than that of DMSO controls, in at least one of the duplicates of 1^st^ screen or in one quarter of the wells in a serial dilution in the 2^nd^ screen, were considered as positive. Animals were scored as dead if immobile and rod-like. For the 1^st^ screening, compounds were tested at 10 μM in duplicates; for the 2^nd^ screening, positive compounds in first screening were tested at 14 different concentrations ranging from 0.000031 to 50 μM. For the third screening, 16 positive compounds were tested on NGM agar plates in a serial dilution manner with the final concentration being that of the compound in the agar. The chemicals were purchased from Sigma-Aldrich (St. Louis, MO), with the exception of cypermethrin, which was purchased from Santa Cruz Biotechnology, Inc (Dallas, TX). Drugs were treated either after (post-treatment) or before (pre-treatment) hypoxia. Post-treatment: synchronized young adult worms were recovered from hypoxia on OP50 plates containing indicated compounds. The compound-containing plates were prepared by freshly smearing with compound dissolved in M9 plus 0.4% DMSO (390 μl in total) onto plates 2h before use. Pre-treatment: synchronized young adult worms were incubated on OP50 plates containing indicated compounds for 24 hours at 20°C and washed off before hypoxia incubation.

### Hypoxic injury scoring

Organismal death was scored as described [11]. Briefly, animals were scored as dead if pharyngeal pumping, spontaneous and evoked movement (touching with a platinum wire) were not observed.

### RNAi experiments

Control RNAi strain, L1440 (empty vector) was from the Ahringer *C*. *elegans* RNAi library (MRC Gene service) [14] and *atfs-1*(RNAi) strain was from Vidal ORF-RNAi *C*. *elegans* RNAi library [15] (a kind gift from Dr. T. Schedl, Washington University in St. Louis, MO). Bacterial clones containing RNAi plasmids were cultured and induced with 0.1% β-lactose in S-Basal 100 mg/ml ampicillin for 24 hr at 23°C [7]. Worms were synchronized on RNAi plates for 3.5 days (N2) or 4.5 days (animals in a *rars-1(gc47)* background) until reaching adulthood; worms not reaching adulthood were excluded.

### Mitochondrial UPR reporter

SJ4100 (*zcIs13*[*hsp-6*::GFP]) was used as a reporter for mitochondrial UPR [16]. L4 animals were incubated on OP50 NGM plates containing indicated drug for 48 hr. The GFP expression were imaged and intensity of fluorescence was determined by ImageJ (NIH).

### Langendorff-perfused heart

The Langendorff-perfused mouse heart trials have been performed as described previously, and the protocol was approved by the University of Washington IACUC (Institutional Animal Care and Use Committee) [17,18]. The mice (C57/Bl6, male, 3–4 month old) were heparinized (heparin, 100 U, i.p.) and euthanized (pentobarbital, 270 mg/kg, i.p.) before surgery. After the chest was cleaned with 70% ethyl alcohol, the thoracic cavity was entered and the heart was rapidly removed. The heart was then cannulated via the ascending aorta and perfused in the Langendorff mode with perfusion solution (118 mM NaCl, 25 mM NaHCO_3_, 5.3 mM KCl, 2 mM CaCl_2_, 1.2 mM MgSO_4_, 0.5 mM EDTA) with metabolic substrates (10 mM glucose and 0.5 mM pyruvate), equilibrated with 95% O_2_ and 5% CO_2_ (pH 7.4) at 37°C. After 30 minutes of baseline perfusion, the perfusion was stopped (no-flow ischemia) for 30 minutes and then resumed for another 60 minutes (reperfusion). The left ventricle (LV) of the heart was separated along the long axis into 6 slices (~1 mm thick each slice) by a heart slicer. The slices were stained by triphenyltetrazolium chloride (TTC) and imaged by a digital camera. The weight of each slice was measured and the LV area and infarct area in each slice was determined by ImageJ software (NIH). The infarct weight was calculated as: (infarct area)/(LV area) x slice weight and the ratio between the total infarct weight and the total LV weight of the 6 slices was expressed as percentage of infarction. To confirm the protective effect of potential candidates, at least two of the trials was carried out by an experimenter blinded to the condition. All compounds were added in the perfusion solutions 30 minutes before the ischemia and throughout the reperfusion period.

### Statistics

Two-sided unpaired or paired, as appropriate, t-tests were used for statistical comparisons. Statistics were calculated using GraphPad Prism 6.01 (San Diego, CA, USA) and Excel 2007 (Microsoft, Redmond, WA). Values are expressed as mean ± SD of at least three independent experiments. A P value of ≤ 0.05 was considered significant. EC_50_ (the half-maximally effective drug concentration) was calculated using GraphPad Prism 6.01 by the equation: Y = Y_min_+(Y_max_-Y_min_)/(1+((x/EC50)^-k)) of at least 5 doses tested where k is the slope and Y_min_ is the lowest death percentage and Y_max_ is the highest percentage of death.

## Results

### Chemical screen identifies six protective compounds against delayed hypoxic organismal death

**Fig 1:**
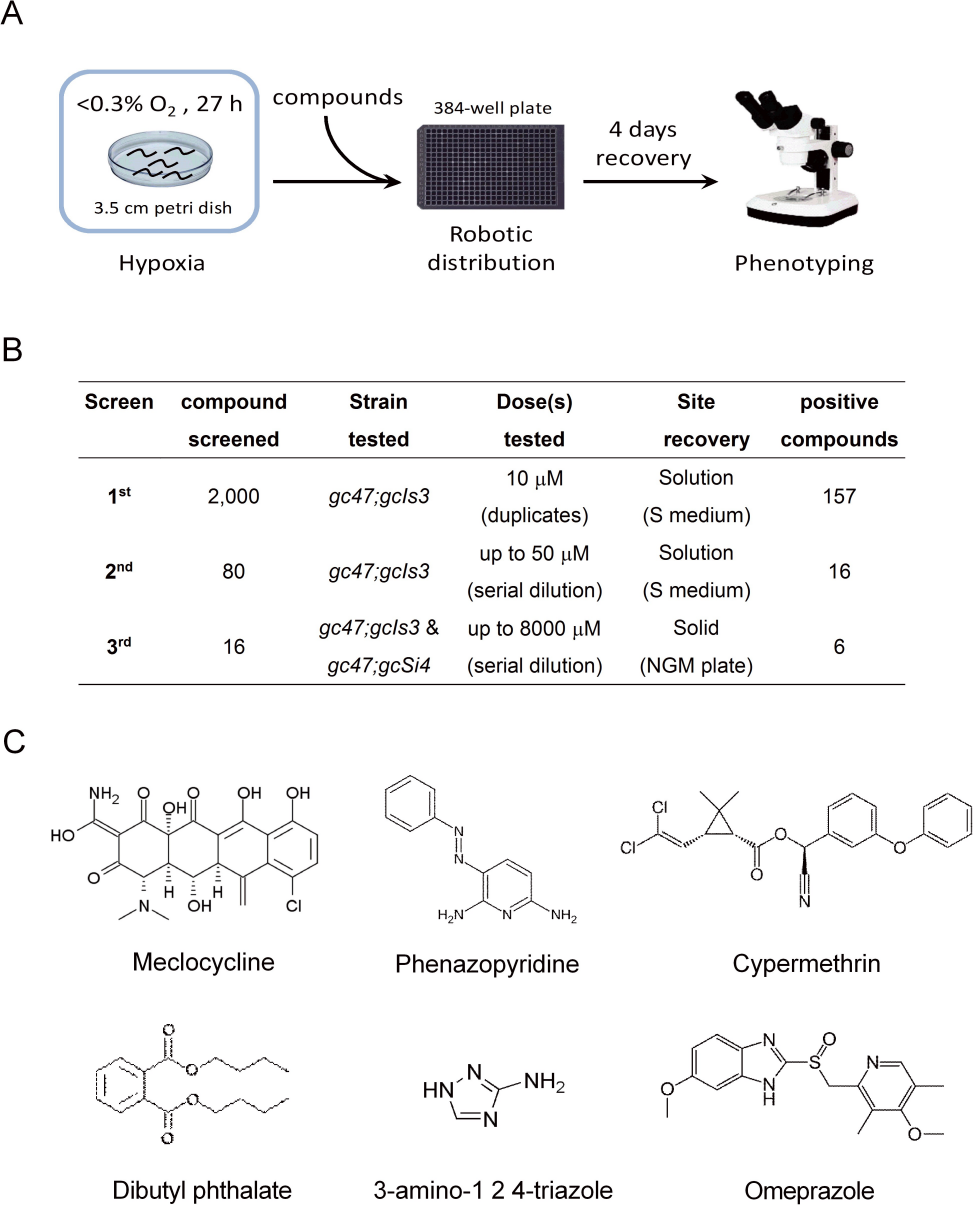
High-throughput screen identifies six compounds protective against delayed hypoxic injuries. (A) The schematic of the screen to identify hypoxic protective compounds. See the Materials and Methods for more details. (B) Summary of three round of screens tested. (C) Chemical structure of six positive compounds identified.

**Fig 2:**
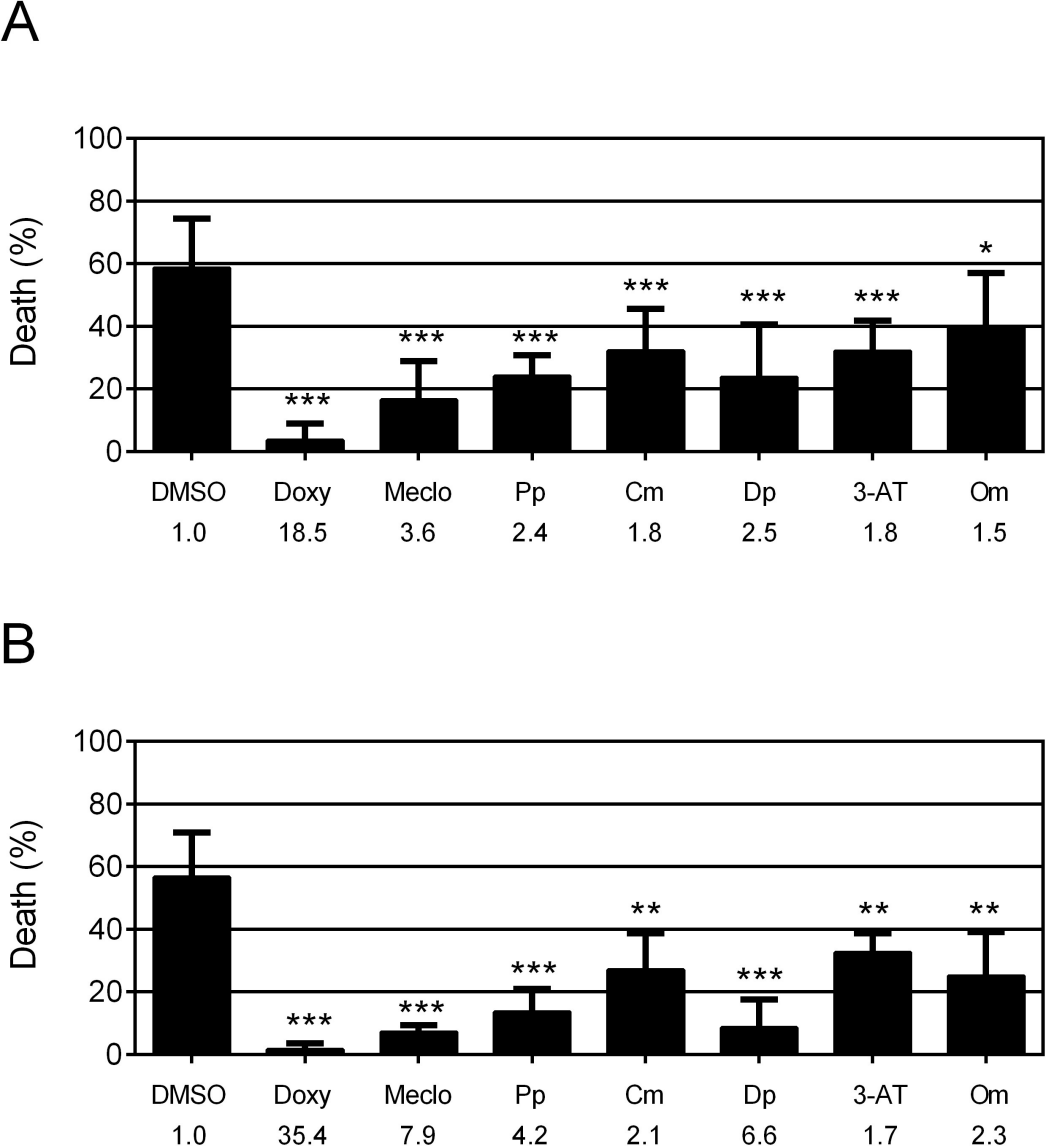
Post-treatment with six compounds protects against delayed focal hypoxic injuries. (A-B) Delayed organismal death of *gc47;gcIs3* (A) and *gc47;gcSi4* (B) following hypoxia and recovery with the various drugs and DMSO in NGM plates. Animals were kept on an individual plate for the entire recovery period then scored. Bars represent mean ± SD (n≥3). P values compared to DMSO control; *, p<0.05; **, p<0.01; ***, p<0.001; unpaired two-tailed *t*-test for mean of the % death. DMSO—dimethyl sulfoxide (solvent negative control, 0.4%); Doxy—doxycycline (positive control, 100 μM) [13]; Meclo—meclocycline (100 μM); Pp—phenazopyridine (50 μM); Cm—cypermethrine (100 μM); Dp—dibutyl phthalate (400 μM); 3-AT—3-amino-1, 2,4-triazole (4000 μM); Om—omeprazole (200 μM). Fold reduction of hypoxic death by drugs compared to DMSO control shown below.

We have previously established a focal hypoxic injury model [11]. The strains used for this model have selective tissue vulnerability to hypoxic injury. *rars-1(gc47);gcIs3* has vulnerable pharyngeal myocytes and *rars-1(gc47);gcSi4* has vulnerable GABA neurons. *gc47;gcIs3* (pharyngeal myocyte, PM-targeted) was used in our initial chemical screen. To mimic a potential therapeutic given after ischemia, drugs were administered to animals during recovery from hypoxic insult. Following recovery, delayed organismal death was scored **(Fig 1A)**. In the 1^st^ screen of the chemical library of 2,000 small molecules, 30 strong and 127 mild protective compounds (157 in total) were obtained. 80 of 157 (30 strong plus 50 mild) potential compounds were subjected to a 2^nd^ screen at multiple drug concentrations. Sixteen compounds (10 of strong compounds and 6 of mild compounds) appeared to have a concentration dependent protection in the 2^nd^ screen and were subjected to a 3^rd^ screen **(Fig 1B)**. The 3^rd^ screen was performed in both *gc47;gcIs3* and *gc47;gcSi4* (GABA neuron, GN-targeted) and unlike the first and second screen, recovery from hypoxia was on agar plates, which is best validated and most reproducible [11]. Compounds with a statistically significant reduction in organismal death of at least 1.5-fold, as compared to DMSO solvent control, were considered positive. Six compounds were confirmed to be significantly protective against delayed hypoxic injury in both the PM-targeted and GN-targeted strains **(Fig 1B and 1C and Fig 2).** The half maximal effective concentration (EC_50_) of the protective compounds ranged from 0.53 to 968 μM **(S1 Fig)**.

### Compounds protect after but not before against focal hypoxic injury

**Fig 3:**
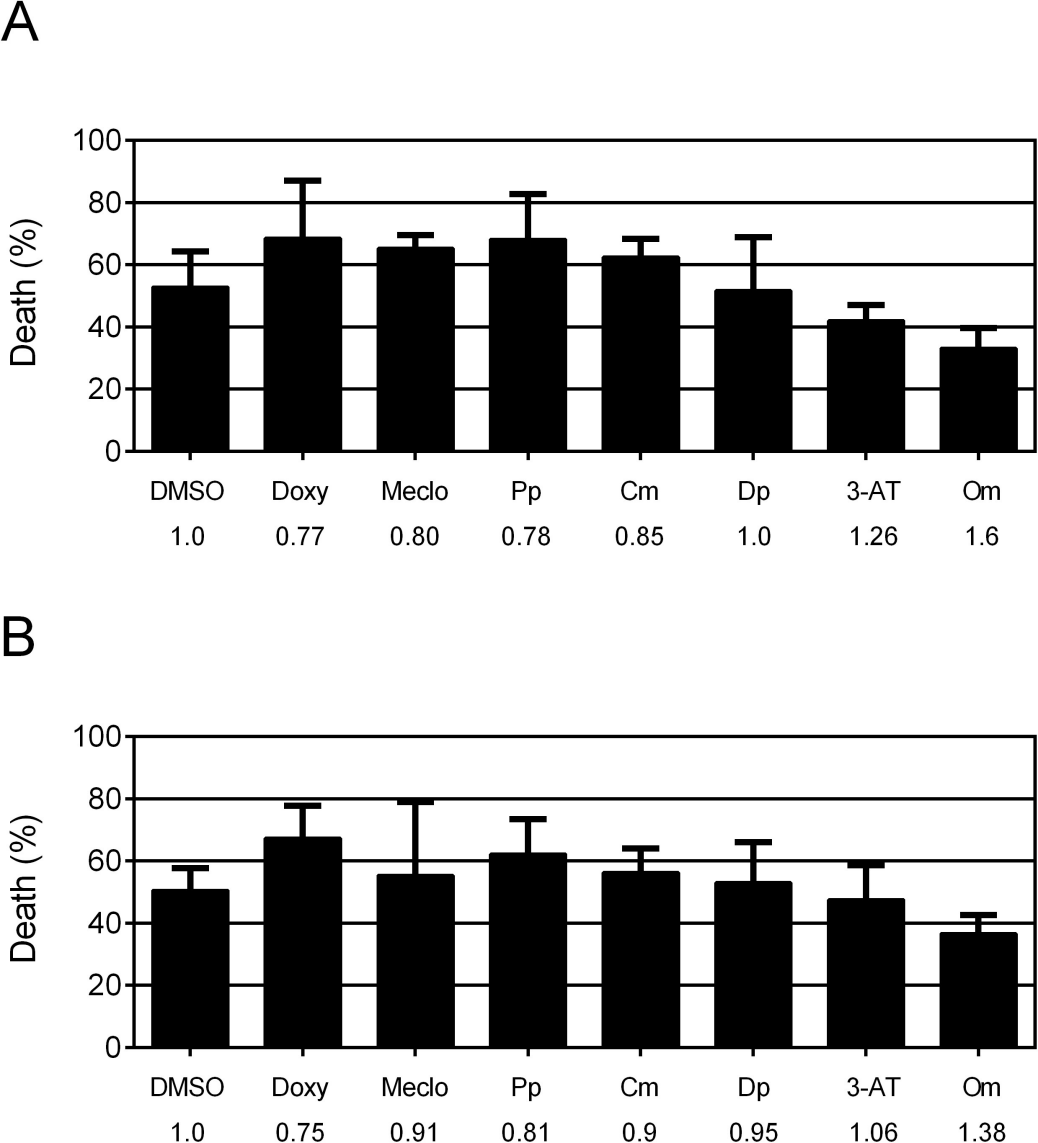
Effect of pre-treatment of six positive compounds on delayed focal hypoxic death. (A-B) Delayed organismal death of *gc47;gcIs3* (A) and *gc47;gcSi4* (B) following hypoxia and recovery. Bars represent mean ± SD (n = 3). None of the values were significantly different from the DMSO negative control at the p < 0.05 threshold. Abbreviations and doses were as described in Fig 2. Fold change in hypoxic death by drugs compared to DMSO control shown below.

We next examined the effect of pre-treatment of the protective compounds on delayed hypoxic organismal death. We reasoned that the protective compounds might confer durable protection to the cells not requiring the continued presence of the compounds. However, none of the six compounds showed protection from delayed hypoxic injury when added for the 24 hours preceding hypoxia in either PM-targeted or GN-targeted strains **(Fig 3)**. These results indicate that underlying mechanisms of those compounds are acting specifically after hypoxic insult.

**Fig 4:**
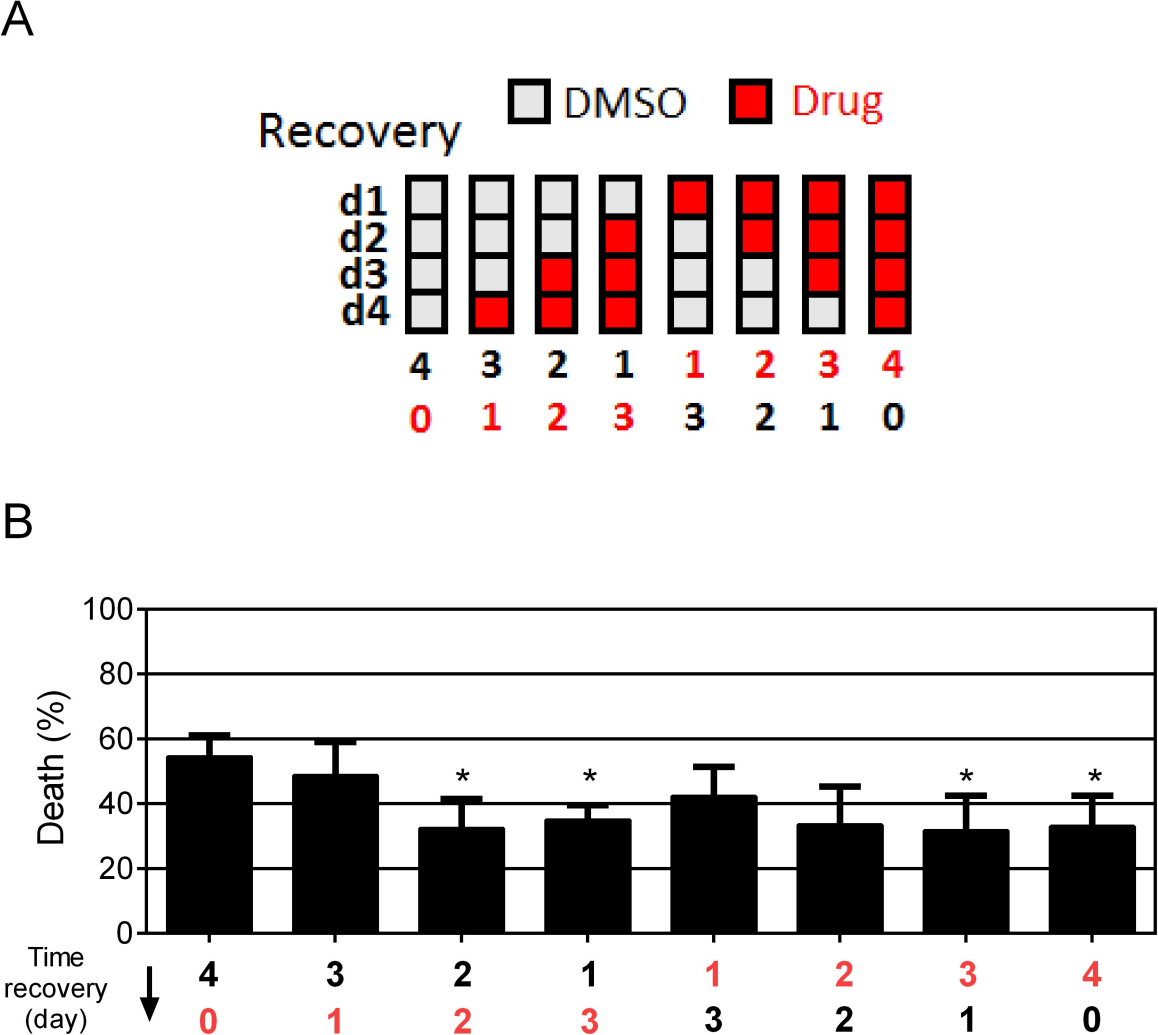
Time course of meclocycline treatment for protection from delayed hypoxic injury. (A) Schematic diagram of meclocycline (Drug) treatment (d1, d2, d3, d4 is the number of days of recovery following hypoxia). Animals were transferred daily to fresh plates containing meclocycline (100 μM) and DMSO (0.15%), as a solvent control. (B) Delayed organismal death of *gc47;gcIs3* following hypoxia with exposure to meclocycline at the indicated recovery intervals. Bars represent mean ± SD (n = 3). P values compared to DMSO control; *, p<0.05 (unpaired two-tailed *t*-test for mean death).

To explore in more detail the timing requirements for improved recovery, we exposed the hypoxic-injured worms to meclocycline at various times during their recovery. Treatment with meclocycline for at least 2 days of the four-day recovery period was required for significant protection and the treatment could be delayed until day 3 and 4 of recovery and still impart a protective effect **(Fig 4)**. These results indicate that meclocycline treatment can improve recovery with a surprisingly delayed time course, up to two days after the initial hypoxic injury.

We next asked whether the compounds were protective in the wild type genetic background where no cells have been genetically modified to increase or decrease their sensitivity to hypoxia relative to their neighbors, thereby modeling global hypoxic injury. None of the compounds protected the wild type strain N2 when added after a short hypoxic exposure that results in a similar level and time course of delayed death as is seen in the *gc47;gcIs3* and *gc47;gcSi4* **(S2A and S2B Fig)**. Notably, phenazopyridine (Pp) appeared to be additive with hypoxia producing injury in the wild type strain suggesting a toxic effect. However, none of the drugs showed toxicity of the adult animals in the absence of hypoxia **(S3 Fig)**.

Likewise, when added before hypoxia, all but one of the compounds failed to protect the wild type strain **(S2C and S2D Fig)**. In contrast, meclocycline as well as its congener doxycycline, when added before hypoxia, were strongly protective in wild type animals **(S2C and S2D Fig)**. Doxycycline and tetracycline were also protective in the focal hypoxic model strains when added after the hypoxic injury (**Fig 2A and 2B and S4 Fig**). Given that these drugs are antibiotics, we considered the possibility that meclocycline and its congeners might be killing or altering the bacteria in some way that indirectly protects *C*. *elegans* from hypoxia. To test this hypothesis, we utilized heat-killed bacteria. Meclocycline, as well as doxycycline and tetracycline, were hypoxia protective when applied with heat-killed bacteria **(S4 Fig)**, indicating that killing or altering the function of the bacteria is not required for protective effect of meclocycline and its congeners.

### mtUPR activation is required for hypoxic protection by meclocycline

**Fig 5:**
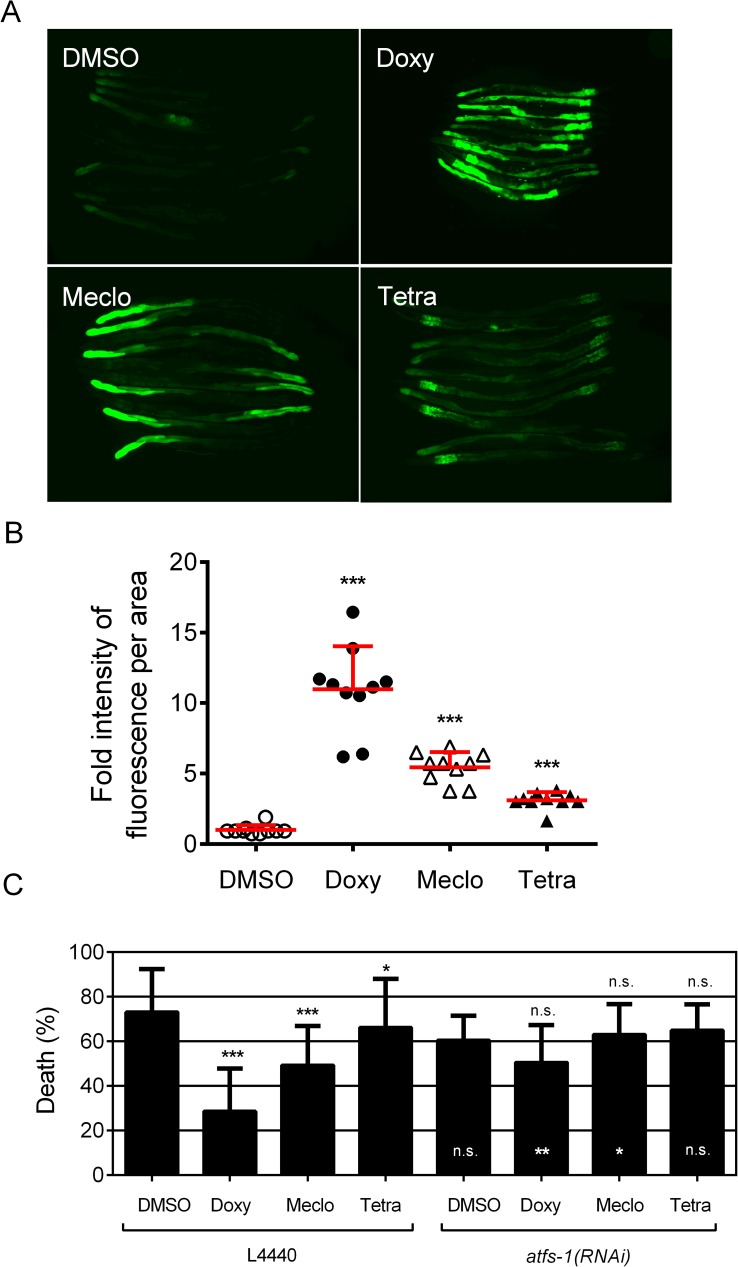
Activation of mtUPR by meclocycline is hypoxia protective. (A, B) Activation of *hsp-6*::GFP by doxycycline, meclocycline, and tetracycline. Two-day exposure of adult animals on plates containing DMSO (0.15%); Doxy, doxycycline (100 μM); Meclo, meclocycline (100 μM); Tetra, tetracycline, (100 μM). (B) Quantitation of *hsp-6*::GFP activation. Fluorescence intensity in arbitrary units/μm^2^ was scored. Data represent mean ± SD (n = 10). P values compared to DMSO control; ***P<0.001 (unpaired two-tailed *t*-test). (C) Effect of *atfs-1* RNAi on meclocycline-mediated delayed hypoxic injuries. Delayed organismal death of *gc47;gcIs3* following hypoxia and recovery with the various drugs and DMSO on empty vector (L4440) or *atfs-1*(RNAi) plates. Bars represent mean ± SD (n = 4). p-values: black, compared to DMSO control; white, *atfs-1*(RNAi) compared to L4440 control for the same drug; n.s., not significant; *, p<0.05; **, p<0.01; ***, p<0.001 (paired two-tailed *t*-test for mean of the % Death). DMSO—dimethyl sulfoxide (solvent negative control, 0.15%); Doxy—doxycycline (positive control, 100 μM); Meclo—meclocycline (100 μM); Tetra—tetracycline (100 μM).

Doxycycline protects *C*. *elegans* in both the global and focal hypoxia injury models via activating mitochondrial unfolded protein response (mtUPR) [13]. We examined whether meclocycline might also protect by activating the mtUPR. Meclocycline treatment induced robust expression of *hsp-6*::GFP, which is a mitochondrial UPR reporter **(Fig 5A and 5B)**. *atfs-1* is a transcription factor central to mtUPR activation [19]. RNAi knockdown of *atfs-1* fully suppressed the protection by meclocycline against delayed hypoxic injury **(Fig 5C)**, indicating that the normal function of the mtUPR is required for the hypoxia protection by meclocycline.

### Meclocycline and 3-AT protect against IR injury in a Langendorff-perfused heart

**Fig 6:**
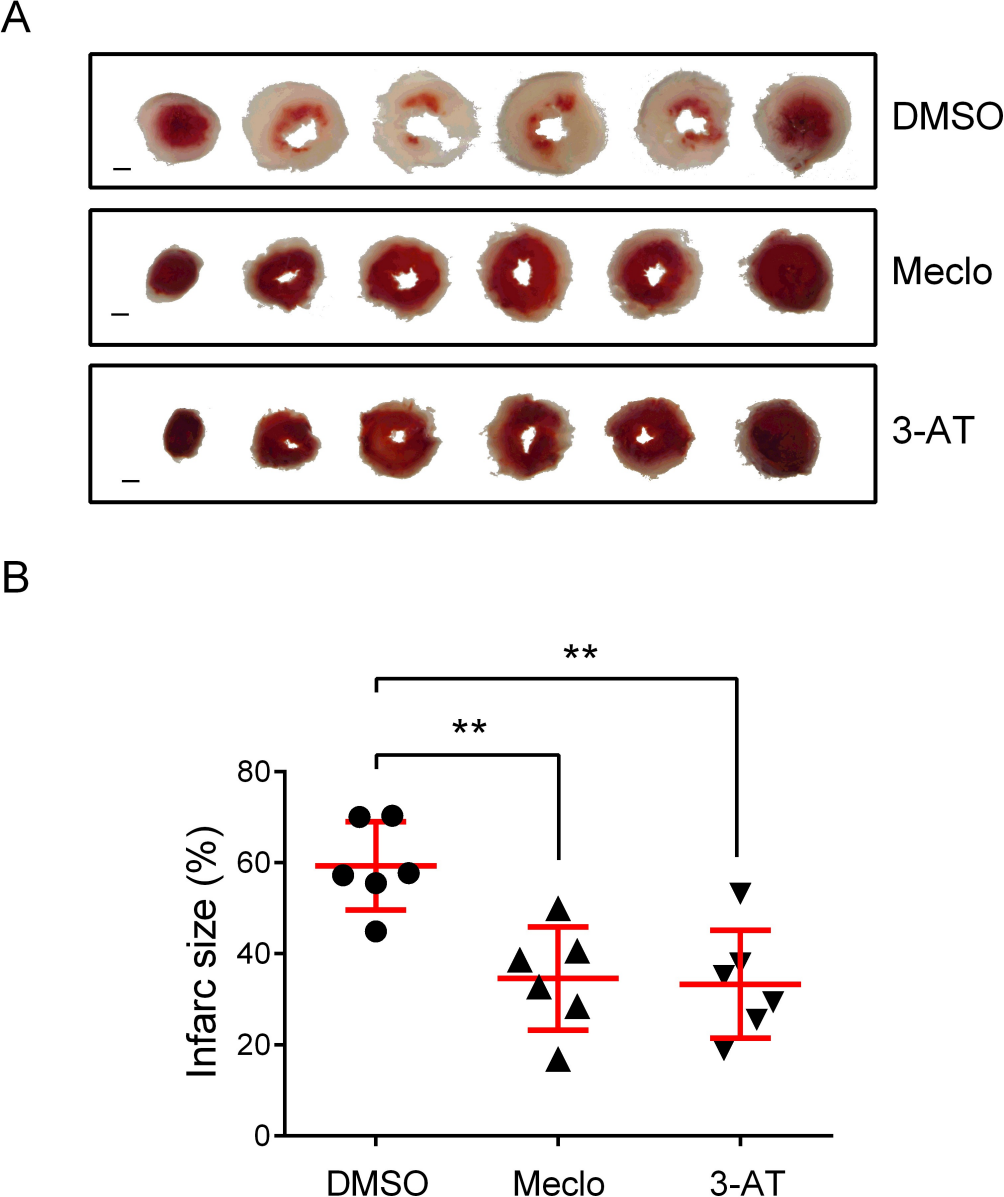
Cardioprotection by two compounds identified from the screen. (A) Examples of representative left ventricular cross-section images (left to right, from bottom to top). DMSO (0.03%), a solvent control; Meclo, meclocycline (20 μM); 3-AT, 3-amino-1,2,4-triazole (600 μM). Bars = 1 mm. (B) Quantitation of infarct size. Data represent mean ± SD (n = 6). P values are by unpaired two-tailed *t*-test compared to DMSO control; **, p<0.01; for meclocycline, p = 0.0023; for 3-amino-1,2,4-triazole, p = 0.0019.

To test whether the protective compounds can protect against IR-mediated hypoxic injury in mammals, we applied the drugs to a Langendorff-perfused mouse heart model. Both the meclocycline- and 3-amino-1,2,4-triazole-perfused hearts were significantly resistant to IR-mediated infarction in comparison with DMSO solvent-perfused controls **(Fig 6)**. By contrast, phenazopyridine-perfused hearts showed no protection and dibutyl phthalate-perfusion was toxic to the heart (data now shown). Cypermethrin and Omeprazole were not tested.

## Discussion

The aim of the current study was to develop a new potentially high throughput screening model for compounds that can improve recovery from hypoxic injury when added after the hypoxic insult. A second goal was to target specifically cell non-autonomous death, which to our knowledge has not been the focus of previous screens, in order to maximize the likelihood of identifying novel therapeutic leads. The potential for novel compounds is particularly valuable here given that a large number of drugs screens for hypoxia protection have been reported and presumably many more have been performed by the pharmaceutical industry without resulting in an approved therapeutic. Using a *C*. *elegans* strain that dies from a delayed cell non-autonomous death, we screened a library of 2,000 chemicals and identified six protective compounds with structural and pharmacological diversity.

Interestingly, none of the six compounds provided protection of the wild type strain when added after the hypoxic insult. In the wild type strain, we believe that cells are more or less similarly sensitive to hypoxia and therefore most closely model a global hypoxic injury paradigm. Whereas in the engineered strain used for the screen, a small number of cells are deliberately sensitized to hypoxic injury so that the primary mode of cell death must be by a secondary, non-autonomous mechanism. Thus, the ability of these six compounds to protect the engineered strain but not the wild type strain when added after the hypoxic injury demonstrates that the mechanisms underlying delayed cell non-autonomous hypoxic death and delayed global hypoxic death are distinct. Also, none of the six compounds protected the engineered strain when present only before the hypoxic insult. However, one of the six compounds, meclocycline, did strongly protect the wild type strain when added only before the hypoxic insult. Doxycycline similarly strongly protected the wild type strain when added before hypoxia. These results show, not surprisingly, that the mechanisms that prevent hypoxic injury are not identical with those that improve recovery after the injury. However, the efficacy of meclocycline and doxycycline both before and after hypoxia in the wild type strain suggests overlap of some aspects of the mechanisms operant in the two scenarios. Given the divergent protective properties and structures of the compounds, a more detailed discussion of the bioactivities of those compound warrants some discussion.

**Cypermethrin**, a synthetic pyrethroid, is commonly used as an insecticide. Cypermethrin is a fast-acting neurotoxin in insects and also induces dopaminergic neurodegeneration in rats [20,21]. Excessive exposure of cypermethrin in humans can cause nausea, headache, muscle weakness, salivation, shortness of breath and seizures [22]. It has been documented that cypermethrin can cause oxidative stress, DNA damage and inhibition of gap junctions, so it is therefore classified as a possible human carcinogen [22–24]. Whether cypermethrin protects from delayed hypoxic injury through any of these known actions remains to be investigated.

**Dibutyl phthalate** is a commonly used plasticizer and also used as an additive to adhesives or printing inks. Exposure of phthalates may cause reproductive and developmental defect [25]. However, how phthalates modulate biological pathways is not understood.

**Phenazopyridine** is a chemical generally prescribed for its local analgesic effects on the urinary tract; it is rapidly excreted by the kidneys directly into the urine [26]. However, the underlying mechanism of phenazopyridine action is not well known. 2,3,6-triaminopyridine, a metabolite of phenazopyridine, causes muscle necrosis and renal damage in rats and promotes generation of superoxide radical and hydrogen peroxide [27,28]. Whether phenazopyridine inhibits delayed hypoxic injury through its metabolite, 2,3,6-triaminopyridine, by regulating generation of reactive oxygen species remains to be determined.

**Omeprazole** is a proton pump inhibitor which blocks the release of stomach acid [29]. In addition, cellular proton levels are strictly controlled and crucial for normal physiology, such as mitochondrial homeostasis [30,31]. How a proton pump, like omeprazole, regulates delayed hypoxic injury is of interest.

**3-amino-1,2,4,-triazole** is characterized as a catalase inhibitor [32] and has been tested for its role in heat shock (hyperthemia)-mediated cardiac injury; however the results were controversial [33,34]. 3-amino-1,2,4,-triazole has also been shown to be neuroprotective in an ischemic brain model [35]. Our data show that 3-AT is also protective against ischemia/reperfusion injury in mouse heart (**Fig 6**). Although ROS has been thought to be a primary cause of IR injury, findings in this study suggest that increased ROS levels by 3-AT and perhaps phenazopyridine may protect from IR mediated heart infarction. Alternatively, an unknown mechanism underlies their protective actions and further investigations are warranted.

**Meclocycline** is a derivative of a tetracycline antibiotic, which blocks bacterial protein synthesis through inhibiting the 30S bacterial ribosomal subunit. In this study, we have demonstrated that meclocycline protects from hypoxic injury in *C*. *elegans* and IR-injury in mouse heart. Tetracycline and its derivatives, such as doxycycline and minocycline, have been shown to protect ischemia/reperfusion injuries in various tissues, including neuronal, liver and myocardium cells, where roles of tetracyclines in anti-inflammation and inhibition of mitochondrial permeability transition for protection against IR injury have been proposed [36–40]. In *C*. *elegans*, we go on to show that meclocycline at the protective concentrations activates the mtUPR and that the hypoxia protection requires the function of the mtUPR transcriptional activator gene *atfs-1* **(Figs 2 and 5)**. Another tetracycline-type antibiotic, doxycycline has been found to be hypoxia protective in *C*. *elegans* and induce the mtUPR, both by an *atfs-1*-dependent mechanism [13,19], therefore arguing that mtUPR activation has therapeutic potential. Activation of the mtUPR by *cco-1(RNAi)* is also protective from hypoxic injury in *C*. *elegans* [13,19]. Tetracycline is also protective from focal hypoxic injury **(Fig 5 and S4 Fig)**. Additional members of this class of drugs may also be protective against cardiac IR injury, and doxycycline, tetracycline, and any other compounds in this family should be tested.

Statins are a class of cholesterol lowing agents that act by inhibiting HMG-CoA reductase, which is the rate limiting enzyme for mevalonate pathway of cholesterol biosynthesis [41]. Activation of the mtUPR is protective against the toxic effects of mevalonate inhibition by statins in *C*. *elegans* [42]. In a chemical screen, Rauthan et al. identified a tetracycline antibiotic, methacycline, as a potent inducer of mtUPR; however, methacycline exhibits only moderate, if any, protection against statins [43]. Methacycline would be another tetracycline-family member that should be tested in both *C*. *elegans* and in mammalian IR models.

Gou et al. have developed a cell-based IR assay for high-throughput drug screens and have identified 37 protective compounds, of which 4 showed cardioprotection in the Langendorff- perfused rat heart [44]. The 37 compounds are completely distinct from the 6 identified in the current study from the same chemical library. As opposed to the Gou et al screen, an essential feature of our screen was that the compounds were applied after the hypoxic insult. Additionally, unlike the Gou et al screen, our screen was performed in a strain where the death of the animal is due to secondary cell non-autonomous hypoxic injury. These two factors may explain the distinct set of protective compounds isolated in the two screens.

Future studies will include screening additional compounds for other novel hits as well as testing in other IR paradigms such as rodent stroke models. Collectively, our findings demonstrate the feasibility and value of our *C*. *elegans* model for identifying compounds that can improve recovery from hypoxic injury when added after the insult.

## Supporting information

S1 Fig EC50 of six protective compounds. (A-F) Dose response curve of protective compounds against hypoxic organismal death in *gc47;gcIs3*. (G) Summary of EC_50_, k and R square of the compounds tested, which were calculated using GraphPad Prism 6.01 (San Diego, CA, USA). Data represent mean ± SD.
(TIF)

S2 Fig Effect of six protective compounds against global hypoxic injury. (A-B) Post-treatment. Wildtype N2 animals were treated with 14 (A) or 16 (B) hr hypoxia followed by 1-day recovery on indicated compound-containing plates. Organismal death scored. (C-D) Pre-treatment. Animals were pre-treated with compounds 24h before hypoxia. Organismal death scored 1 day after a 14 (A and C) or 16 (B and D) hr hypoxia. Bars represent mean ± SD (n = 3). Abbreviations were as described in Fig 2. P values compared to DMSO control; ***, p<0.001; paired two-tailed *t*-test for mean of the % death.
(TIF)

S3 Fig Test for toxic effects of positive compounds. (A, B) Delayed organismal death of *gc47;gcIs3* (A) and *gc47;gcSi4* (B) following 27h normoxia at 26.5°C and 4 days recovery (n = 1). Abbreviations and doses were as described in Fig 2.
(TIF)

S4 Fig Protective effect of tetracyclines is independent of bacterial survival. (A, B) *gc47;gcIs3* (A) or *gc47;gcSi4* (B) were fed before and after hypoxia with either live or heat-killed OP50. Delayed organismal death (death) scored by 4-day recovery following hypoxia. Bars represent mean ± SD (n = 3). P values are by unpaired two-tailed *t*-test compared to DMSO control; *, p<0.05; n.s., not significant. DMSO (0.15%) as a solvent control; Doxy—doxycycline (100 μM); Meclo—meclocycline (100 μM); Tetra—tetracycline (100 μM).
(TIF)
